# Immunosensors for Autoimmune-Disease-Related Biomarkers: A Literature Review

**DOI:** 10.3390/s23156770

**Published:** 2023-07-28

**Authors:** Chrysoula-Evangelia Karachaliou, Evangelia Livaniou

**Affiliations:** Immunopeptide Chemistry Lab, Institute of Nuclear & Radiological Sciences & Technology, Energy & Safety, National Centre for Scientific Research ‘‘Demokritos”, P.O. Box 60037, 153 10 Agia Paraskevi, Greece; xrisak15@hotmail.com

**Keywords:** autoantibodies, autoimmune diseases, patients’ biological fluids, immunosensors, inflammation, protein-biomarkers

## Abstract

Immunosensors are a special class of biosensors that employ specific antibodies for biorecognition of the target analyte. Immunosensors that target disease biomarkers may be exploited as tools for disease diagnosis and/or follow-up, offering several advantages over conventional analytical techniques, such as rapid and easy analysis of patients’ samples at the point-of-care. Autoimmune diseases have been increasingly prevalent worldwide in recent years, while the COVID-19 pandemic has also been associated with autoimmunity. Consequently, demand for tools enabling the early and reliable diagnosis of autoimmune diseases is expected to increase in the near future. To this end, interest in immunosensors targeting autoimmune disease biomarkers, mainly, various autoantibodies and specific pro-inflammatory proteins (e.g., specific cytokines), has been rekindled. This review article presents most of the immunosensors proposed to date as potential tools for the diagnosis of various autoimmune diseases, such as type 1 diabetes, rheumatoid arthritis, and multiple sclerosis. The signal transduction and the immunoassay principles of each immunosensor have been suitably classified and are briefly presented along with certain sensor elements, e.g., special nano-sized materials used in the construction of the immunosensing surface. The main concluding remarks are presented and future perspectives of the field are also briefly discussed.

## 1. Introduction

Autoimmune diseases (ADs) are a group of various disorders that are characterized by dysregulation of the immune system. This malfunction state leads to an improper activation of immune elements that may consequently attack target molecules, cells, and tissues of the organism, resulting in inflammation and organ damage [1]. ADs are considered chronic disorders that develop over several years [2] and may be divided into organ-specific or multi-organ/systemic [3]. Thus, in type 1 diabetes (T1D) [4], an organ-specific AD, the autoimmunity-related inflammatory responses of the organism are specifically directed against the β-islet cells of the pancreas, which are eventually destroyed, leading to insulin deficiency. By comparison, in rheumatoid arthritis (RA) [5], a multi-organ/systemic autoimmune disease, inflammation is “dispersed” to the joints of the organism, as the synovial membrane is infiltrated by activated immune cells. According to the literature [6], some ADs exhibit both systemic and organ-specific characteristics; for instance, multiple sclerosis (MS) [7] is characterized by inflammation in the brain and spinal cord, which results in neuron axon and myelin damage in the central nervous system, and, consequently, general disturbances [6]. Usually, ADs follow a pattern of progressively increased severity, while the insufficiency of the treatment protocols available at present, combined with autoimmune attack of vital organs, such as lungs and kidneys, may eventually lead to a fatal outcome [1].

ADs are currently estimated to affect almost 5–7% of the world population, while it is well accepted that their prevalence will continue to rise worldwide in the following years [6]. The etiology of ADs is basically unknown, but it has mainly been associated with genetic and epigenetic factors along with environmental ones, such as nutritional habits and air pollution [6]. Moreover, it is widely accepted that specific viral infections can cause development of ADs [6]. For instance, infection with Epstein–Barr virus has been associated with the pathogenesis of RA [8] and MS [9]. Furthermore, several literature reports associate the SARS-CoV-2 virus, which caused the recent pandemic, with development of autoimmunity/autoantibodies [10,11,12]. Nevertheless, the exact mechanisms by which certain viruses may trigger the onset of ADs have not yet been well elucidated.

### Biomarkers and Analytical Tools

ADs are closely associated with the generation of autoantibodies against certain self-antigens that can subsequently attack cells, tissues, and organs of the organism. Many autoantibodies have been associated with pathogenesis/treatment of specific ADs, such as systemic lupus erythematosus (SLE) [13]. Moreover, a series of autoantibodies has been proposed to serve as diagnostic biomarkers for ADs [14,15,16]; specifically, various anti-citrullinated protein/peptide antibodies (ACPAs) and rheumatoid factors (RFs), i.e., IgM, IgA, and IgG antibodies directed against the Fc fragment of the patient’s own IgG molecules, were included in the classification criteria for RA set by the European League against Rheumatism (EULAR)/American College of Rheumatology (ACR) [17,18,19].

Production of inflammatory protein mediators is considered a common feature of ADs. Thus, pathogenesis of RA has been closely related to various inflammatory proteins, including tumor necrosis factor-α (TNF-α), interleukin-1β (IL-1β), and interleukin-6 (IL-6), while several interleukins, including interleukin-1 (IL-1), IL-6, interleukin-8 (IL-8), interleukin12 (IL-12), and interleukin-32 (IL-32), have been proposed as promising biomarkers of a series of rheumatic ADs [20,21,22,23,24]. Other protein-biomarkers have been associated with diagnosis/prognosis/follow-up of specific ADs; for instance, the protein hormone insulin has been correlated with the diagnosis of various types of diabetes and its low circulating levels have been proposed to signify T1D [25].

All the above-mentioned AD biomarkers are of a protein nature; however, microRNAs, extracellular vesicles/exosomes, etc., have also been proposed for the diagnosis of specific ADs, such as RA, MS, and T1D [26,27,28].

Due to the wide prevalence and severity of symptoms of ADs, demand for analytical tools detecting AD-related biomarkers and enabling early and reliable diagnosis of the disease state is very high and expected to further increase in the near future. To date, the methods most frequently used for determining AD biomarkers of a protein nature are immunoassays and, in particular, ELISAs, while other biochemical assays, such as Western blot, have been also reported. The disadvantages of these methods often include low sensitivity, along with time-consuming, laborious, and complex protocols; moreover, most of these methods require skilled personnel and well-equipped laboratories. Therefore, during the last two decades, attention has focused on developing biosensors that are specific, sensitive, reproducible, and stable, as well as simple, fast, and low-cost, for the determination of protein-type AD biomarkers in complex clinical samples, such as blood sera, preferably at the point of care [1,29].

Biosensors are analytical devices that employ specific biomolecules for recognizing the target entity; biosensors integrate the biorecognition element with suitable transducers, which could be further enriched with various signal amplification systems to achieve extremely high sensitivity. Immunosensors are a special class of biosensors that employ specific antibodies as the biorecognition part; upon specific recognition and capture of the target analyte, a series of physical and/or chemical changes is induced, which could be converted into detectable signals by an appropriate transducer.

Depending on the type of transducer employed, biosensors/immunosensors can be characterized as electrochemical, optical, piezoelectric, or thermal [1,24,29,30,31,32]. In electrochemical biosensors/immunosensors, recognition and binding of the target analyte to its specific partner biomolecule usually causes a change in the electron transfer rate between a properly functionalized electrode surface and an electrolyte solution; this change can be detected as a current (amperometric sensors), charge accumulation or potential (potentiometric sensors), or change in conductivity (conductimetric/impedimetric sensors). Electrochemical biosensors/immunosensors have many advantages, including high analytical sensitivity and specificity, rapid assay protocol, low quantity of sample needed, and potential of developing miniaturized devices for point-of-care analyses. In optical biosensors/immunosensors, recognition/binding of the target analyte to the partner biomolecule causes measurable changes in the phase, amplitude, polarization, or frequency of the input light. Optical immunosensors include colorimetric, fluorescent, luminescent, and reflectometric/refractometric sensors [31]. Optical biosensors/immunosensors also have a series of desired characteristics, such as high sensitivity, high specificity, and rapid assay protocol, but their instrumentation is often bulky and expensive; nevertheless, much work has been recently devoted to address these limitations and enhance optical sensors’ applicability. In piezoelectric biosensors/immunosensors, recognition/binding of the target analyte usually causes a mass change on the sensing surface of the device. These sensors, which are usually based on quartz crystal microbalance (QCM) resonators, are not as widely used as the electrochemical and optical ones, mainly because of the complexity of their assay protocol. Thermal biosensors are based on the heat energy absorbed or released in biochemical reactions [32] and have very rarely been used in the field of diagnosis. It should be noted that various nanomaterials with desired features have been employed in the design/construction of biosensors/immunosensors, especially electrochemical and optical ones, aiming at further improving the analytical sensitivity and specificity of the latter [33,34,35].

Several review articles have presented biosensors/immunosensors for ADs, some of which deal only with electrochemical sensors, while others present information on sensors based on more types of signal transduction [1,3,24,36]. Some recent reviews have described biosensors/immunosensors proposed to serve diagnosis of specific ADs, such as MS [37,38] and RA [39], while other review papers have focused on biosensors/immunosensors for the detection of specific AD-related biomarkers, such as IL-6 [23].

**Figure 1:**
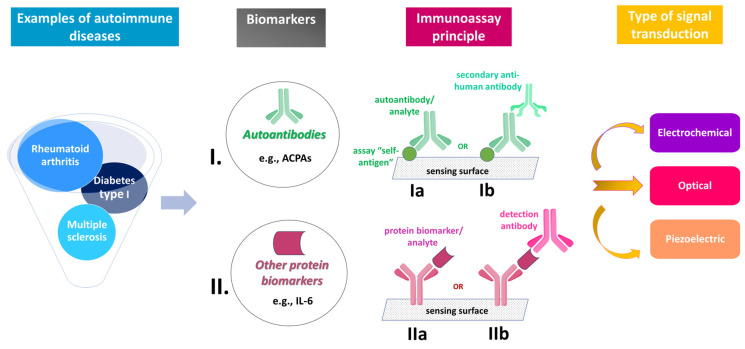
Schematic representation of the main principles on which immunosensors for autoimmune diseases (AD immunosensors) developed to date are based. The sensors can be divided into two broad categories, i.e., those detecting autoantibodies (**I**) and those detecting other protein biomarkers (**II**). All sensors are based on a non-competitive immunoassay principle. The sensors of group I follow a direct-type immunoassay setting (**Ia**), in which an anti-human secondary antibody may also be used (**Ib**). The sensors of group II may follow either a direct-type (**IIa**) or a sandwich-type (**IIb**) immunoassay setting; in the latter, a couple of capture and detection anti-analyte antibodies (often along with a secondary antibody, for signal enhancement) are employed. Depending on their signal transduction principle, the AD immunosensors may be classified as electrochemical, optical, or piezoelectric.

In this work, we have attempted to compile and present the vast majority of immunosensors for ADs (AD immunosensors), focusing especially on the most recently reported ones (from 2019 to now), which may have not been presented in detail in previous review articles. Moreover, in comparison with the previous reviews, in this work we placed more emphasis on the immunoassay principle of the AD immunosensors (Figure 1), and tried to present a general overview of the field, including all types of sensors, independently of the signal (electrochemical, optical, or piezoelectric) generated/measured in response to the basic immunochemical interaction, or the specific AD/AD biomarker the sensors have been developed for. For practical reasons, AD immunosensors have been divided into two main categories depending on the biomarker(s) detected, i.e., either various autoantibodies or other protein biomarkers, such as specific cytokines (Figure 1).

## 2. AD Immunosensors Detecting Autoantibodies

AD immunosensors detecting autoantibodies are mainly electrochemical, although some optical and piezoelectric-type sensors have been reported. Concerning the immunoassay principle, all sensors are non-competitive and based on the direct binding between an appropriate derivative of the “self-antigen” and the relevant autoantibody, which is actually the assay analyte. Keeping this in mind, one should note that, in a way, all affinity-based biosensors that detect autoantibodies can, by definition, be considered as immunosensors, i.e., even if they do not employ “external” antibodies as assay reagents. In many sensors, however, a secondary antibody is indeed employed, either labeled or not, which is added to the predominant immunocomplex, mostly to enhance the assay signal. In such immunosensors, the actual immunoassay setting is antigen–autoantibody/analyte–secondary antibody.

Regarding the most recently reported AD immunosensors that can detect autoantibodies (from 2019 to now), some points to note are discussed below in brief:

An electrochemical immunosensor for autoantibodies against oncostatin-M receptor (OSMR), which has been associated with the AD known as systemic sclerosis [40], was developed. The sensor was based on tin dopped indium (ITO) electrodes coated with a conductive layer of poly-pyrrole (PPy); PPy was suitably loaded with gold nanoparticles, on which OSMR was immobilized, so as to capture the corresponding autoantibodies [41]. ACPAs, a well-known biomarker of RA, as already mentioned, were detected by an electrochemical immunosensor using interdigitated electrodes, the surfaces of which were suitably modified with a cyclic citrullinated peptide loaded on iron oxide nanoparticles. The nanoparticles were synthesized with a “green-chemistry” approach based on a plant extract and chemically immobilized on the sensing surface by means of 3-aminopropyltriethoxysilane and glutaraldehyde [18]. Another electrochemical immunosensor for the detection of ACPAs was based on electrodes, the surfaces of which were functionalized with avidin. Avidin was covalently immobilized on the sensing surface, which had been covered with a self-assembled monolayer of mercaptohexanoic acid through N-hydroxy succinimide/1-ethyl-3-(3-diethylamino)propyl carbodiimide. A biotinylated cyclic citrullinated peptide was bound on the functionalized surface, which could subsequently capture/detect any anti-citrullinated peptide/protein autoantibodies present in the biological sample [42]. A customized quartz crystal microbalance (QCM) point-of-care immunosensor was developed for autoantibodies against the phospholipase A2 receptor (anti-PLA2R), which has been reported to serve as a biomarker in primary membranous nephropathy [43]. The sensor was based on QCM chips coated with reduced graphene oxide; the sensing surface was functionalized through bovine serum albumin adsorption, activation with N-hydroxysuccinimide/1-ethyl-3-(3-diethylamino)propyl carbodiimide, and covalent immobilization of the dominant epitope of PLA2R on the activated surface, which could subsequently capture the anti-PLA2R autoantibodies present in samples to be analyzed [44]. A multiplex, label-free optical immunosensor was developed on microarray glass biochips and applied to both detection and activity characterization of several autoantibodies in the same biological sample. Simultaneous detection of two antibodies that have been associated with thyroid ADs, and especially Hashimoto’s thyroiditis [45], i.e., anti-thyroglobulin (anti-TG) and anti–thyroid peroxidase (anti-TPO) model autoantibodies, was reported. As already mentioned, the sensor could be applied to both quantitating the autoantibodies and evaluating binding kinetics with the corresponding free self-antigen, thus providing more comprehensive diagnostic information [46]. An immunosensor based on impedimetric-interdigitated wave type microelectrode array (IDWμE) was developed and applied to the detection of the rheumatoid factor-immunoglobulin M (IgM-RF) autoantibodies. The IDWμE sensing surface was first functionalized through a self-assembled monolayer of thioctic acid, on which IgG-Fc fragments were covalently immobilized and served as specific “self-antigens”/binders to capture the IgM-RF analyte. In the presence of a suitable redox probe, impedimetric measurements showed a significant change upon IgM-RF binding [47]. An optical immunosensor for IgM-RF was based on gold nanoparticles, on which IgG-Fc was covalently linked through a bi-functional polyethylene glycol derivative. In the presence of pentameric IgM-RF, extensive aggregation of functionalized gold nanoparticles occurred, which resulted in a color change [48]. An immunosensor for the detection of IgG-type autoantibodies against tissue transglutaminase (anti-tTG), which is a reliable serological marker of the gluten-associated AD known as celiac disease [49], was described. The sensor was based on membrane-templated gold nanoelectrodes of composite nature. On the polycarbonate part of the sensor surface used to prepare the nanoelectrode ensembles, the tTG-antigen was immobilized and subsequently used to capture the corresponding autoantibodies. The immune complex was finally electrochemically detected through an HRP-labeled secondary antibody, in the presence of the H_2_O_2_/hydroxyquinone system [50]. “Sick” red blood cells that bear specific autoantibodies and are present in blood of patients with autoimmune hemolytic anemia [51] have been detected by means of an electrochemical immunosensor [52], which might provide more information in comparison with that revealed by other serological diagnostic tests for this AD [53]. Finally, an electrochemiluminescent (ECL) immunosensor was reported for the detection of autoantibodies against myeloperoxidase (anti-MPO), which are present in patients with an AD known as antineutrophil cytoplasm antibody (ANCA)-associated vasculitides [54]. In this sensor, human MPO was immobilized onto glassy carbon electrodes suitably functionalized with gold/MoS_2_ nanosheets, and used to capture mouse anti-human MPO (model autoantibody/analyte). Afterward, a secondary antibody loaded on PtCo nanocubes/reduced graphene oxide hybrids, along with a thiol-modified single-stranded DNA, which triggered a hybridization chain reaction (HCR) to form double-stranded DNA that intercalated doxorubicin/N-(aminobutyl)-N-(ethylisoluminol) (ABEI) luminophores, was used to detect the immunocomplexes formed, through ECL signal generation and amplification in the presence of H_2_O_2_ [55].

Table 1 presents most of AD immunosensors reported to date that can detect autoantibodies in various samples.

Several immunosensors detecting antibodies against the wheat grain protein, gliadin [70,71,72], have often been characterized as AD immunosensors, because the anti-gliadin antibodies can serve as CD biomarkers, as anti-tTG autoantibodies do. Strictly speaking, however, anti-gliadin antibodies may not be considered autoantibodies, since gliadin is a food component, not a mammalian self-antigen.

In addition to the aforementioned AD immunosensors, sensors based on similar technological approaches that can detect cancer-related autoantibodies, such as anti-p53 antibodies, have been developed [73]. Comprehensive reviews focusing on biosensors/immunosensors for AD- and cancer-related autoantibodies have been recently published [1,15].

## 3. AD Immunosensors Detecting Other Protein Biomarkers

Almost all AD immunosensors detecting specific protein biomarkers, other than autoantibodies, are electrochemical, while they are all based on a non-competitive assay principle, of either direct- or sandwich-type. Regarding the most recently reported AD immunosensors (from 2019 to now) of this category, the following can be summarized:

An optical immunosensor was developed for the simultaneous determination of procalcitonin (PCT) and IL-6, which are well-known biomarkers of inflammatory diseases, including ADs. Silicon chips with silicon dioxide areas of different thickness were used, each functionalized with either anti-PCT or anti-IL-6 capture antibodies. Moreover, biotinylated detection antibodies were used along with streptavidin and biotinylated bovine serum albumin, to achieve amplification of the optical signal, in a sandwich-type immunoassay setting [74]. Earlier, an electrochemical immunosensor for IL-6 was developed. The sensor was based on a working electrode modified with a hybrid of gold nanoparticles (AuNPs) and graphene. Magnetic beads loaded with capture anti-IL-6 antibodies were also used, on which IL-6 could be bound and subsequently detected through biotinylated anti-IL-6 antibodies (sandwich-type assay setting) and HRP-labeled streptavidin [22]. An electrochemical immunosensor for oncostatin-M receptor, a soluble form of which (sOSMR) had been found at increased levels in sera of patients with systemic sclerosis, was based on a conductive poly-pyrrole layer loaded with gold nanoparticles, on which anti-sOSMR antibodies were immobilized through cysteine chemisorption [41]; this sensor has been already mentioned (i.e., in “2. AD Immunosensors Detecting Autoantibodies”), since, after a slight modification of the assay format/protocol, it may also be applied to the detection of autoantibodies against sOSMR [41]. Another electrochemical immunosensor was developed for simultaneous determination of the cytokines B-cell activation factor (BAFF) and a proliferation-induced ligand (APRIL), both of which have been associated with systemic lupus erythematosus (SLE) [75]. Biotinylated anti-BAFF and anti-APRIL capture antibodies were loaded onto magnetic beads through neutravidin or direct covalent immobilization, respectively, while detection anti-BAFF antibodies along with HRP-labeled secondary antibodies, and detection biotinylated anti-APRIL antibodies along with HRP-labeled streptavidin, were used for assaying BAFF and APRIL, respectively [76]. In another electrochemical immunosensor for simultaneous determination of BAFF and APRIL, biotinylated anti-BAFF and biotinylated anti-APRIL capture antibodies were indirectly immobilized onto the working electrodes, on which neutravidin had been covalently bound. A sandwich-type assay setting was achieved by using anti-BAFF and anti-APRIL detection antibodies along with HRP, all of which were loaded onto nanostructures composed of MoS_2_/multiwall carbon nanotubes (MoS_2_/MWCNTs), which enabled signal generation (through the hydroquinone/H_2_O_2_ system) and amplification [77]. An electrochemical immunosensor for the MS-associated protein biomarker chemokine (C-C motif) ligand 5 (CCL5) was developed. CCL5 was captured from biological samples through biotinylated anti-CCL5 antibodies (immobilized onto magnetic microparticles pre-functionalized with neutravidin) and subsequently detected with anti-CCL5 antibodies along with HRP-labeled secondary antibody (through the amperometric signal produced in the presence of H_2_O_2_ and using hydroquinone as the redox mediator) in a sandwich-assay setting [78]. One more electrochemical immunosensor has been developed that can serve simultaneous determination of the CXCL7 chemokine and the MMP3 enzyme/metalloproteinase, both of which are present at elevated levels in serum of RA patients. The sensor has been based on capture anti-CXCL7 and anti-MMP3 antibodies covalently immobilized on magnetic beads and on detection biotinylated anti-CXCL7 and anti-MMP3 antibodies along with HRP-streptavidin conjugates [79]. Some years earlier, an electrochemical immunosensor was developed for detecting TNFα, the well-known inflammatory protein biomarker; the sensor was based on a gold working electrode functionalized with anti-TNFα antibodies through covalent immobilization by means of sulfosuccinimidyl 6-(3′-(2-pyridyldithio) propionamido) hexanoate (sulfo-LC-SPDP) [80].

It should be noted that the term “immunosensor” is sometimes used in the literature to define a properly modified specific antibody recognizing/”sensing” a protein biomarker, such as TNF-α [81]; such antibodies (“quenchbodies”) can be used in various applications, e.g., in conventional immunoassays.

In Table 2 we have attempted to include most of the AD immunosensors reported to date that detect specific protein biomarkers, other than autoantibodies, and present their main characteristics.

In addition to the inflammation/AD-related protein biomarkers shown in Table 2, a recent review article has presented various analytical assays for HMB1 [87]. HMB1 is a well-known multifunctional protein associated with severe inflammation storms and proposed as a prognostic biomarker for COVID-19; an electrochemical immunosensor prototype for detecting its levels in fluids has been described in this article [87].

In addition to the AD immunosensors, AD aptasensors have been developed for the detection of biomarkers, such as TNFα, various interleukins, and C-Reactive Protein [88,89]. In these AD aptasensors, the specific anti-analyte antibodies were replaced with especially designed and prepared binders, known as aptamers [90]. Moreover, sensors based on molecularly imprinted polymers have been reported in the literature, e.g., for the detection of insulin [91].

## 4. Discussion

Due to the continuously increasing number of patients with ADs, the severity of ADs’ clinical symptoms, and treatment insufficiency, novel analytical tools enabling early, reliable, and high-throughput disease diagnosis are highly desirable, since such tools may help health systems to confront the burden related to the late diagnosis of ADs and decrease premature mortality. To meet this need, during the last two decades, several immunosensors for detecting AD-related biomarkers have been developed as research prototypes.

The AD immunosensors reported to date can be divided into two main groups, depending on the biomarkers they detect, i.e., autoantibodies or other types of protein markers (Figure 1). All of them are based on a non-competitive (direct or sandwich) immunoassay principle (Figure 1), at least to the best of our knowledge; while the vast majority of these are electrochemical, some of them are optical and a few are piezoelectric.

**Figure 2:**
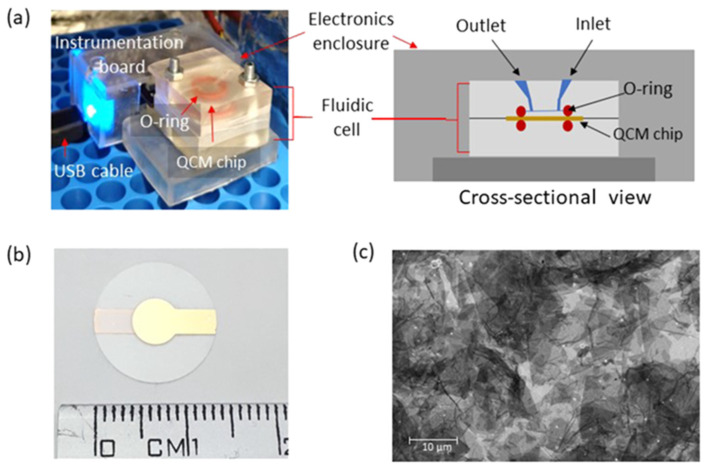
Photos, schematics, and images of a piezoelectric immunosensor developed for detecting a pMN-associated autoantibody biomarker, i.e., anti-phospholipase A2 receptor autoantibody (anti-PLA2R autoantibody). (**a**). Instrument photo and cross-sectional schematic view of an in-house-developed QCM device with a custom-designed microfluidic channel. (**b**). Photo of a QCM sensing chip coated with reduced graphene oxide (rGO). (**c**). Scanning electron microscopy (SEM) of the rGO coating on the QCM chip. (Reproduced from [44]).

**Figure 3:**
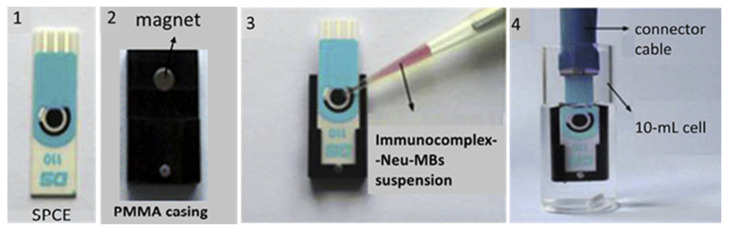
Photos of an electrochemical immunosensor developed for detecting an MS-associated protein biomarker, i.e., CCL5 chemokine. The sensor was based on biotinylated capture anti-CCL5 antibodies immobilized on magnetic beads (MBs) pre-coated with neutravidin (Neu), which could form sandwich-type immunocomplexes with the CCL5 analyte, detection anti-CCL5 antibodies and, finally, horseradish peroxidase-labeled secondary antibodies; the electrochemical signal is generated in the presence of the H_2_O_2_/hydroquinone system. (**1**). SPCE: Screen-printed carbon electrode. (**2**). Magnet casing prepared in-house from polymethylmethacrylate (PMMA) with an encapsulated magnet. (**3**). Addition of the suspension of MBs-Neu-immunocomplexes onto the SPCE-sensing surface. (**4**). SPCE with the MBs-Neu-immunocomplexes immersed into a 10 mL electrochemical cell to measure the electrochemical signal. (Reproduced from [78]).

Most of the AD immunosensors reported to date have been applied to the analysis of human samples, especially blood serum, with very promising results (Table 1 and Table 2). Since complex biological samples contain substances that may cause non-specific binding and thus affect assay results, the high assay specificity and sensitivity provided by the AD immunosensors are considered very important. Actually, the LoD values achieved with AD immunosensors are often remarkably improved as compared with those of the corresponding well-established ELISA methods; for instance, an LoD of 0.42 pg mL^−1^ has been achieved with the electrochemical AD-immunosensor detecting the SSc-associated AD biomarker, oncostatin M receptor (OSMR), instead of the 312.50 pg mL^−1^ LoD value reported for the corresponding gold-standard ELISA method [41]. These highly desired analytical features may be attributed to particular procedures followed during the immunosensors’ design/construction. The latter include special chemical modifications and functionalization of the sensing surface so as to improve the efficiency of biorecognition and the generation/amplification of specific vs. non-specific signals. To this end, a series of blocking materials, most of which are also widely used in conventional immunoassays, e.g., bovine serum albumin, polyethylene glycol, glycerol, ethanolamine, and Tween-20, have been used to prevent non-specific interactions on the sensing surface, or as assay-buffer ingredients. Other chemicals, e.g., hydroxyl- or carboxylic terminated self-assembled monolayers of alkanethiol, have been used to improve the ratio of specific vs. non-specific signals, thus enhancing the assay sensitivity and specificity. On the other hand, various novel materials, such as suitably modified magnetic beads and nanoparticles, along with other specially designed nanostructures (properly functionalized gold nanoparticles, multi-walled carbon nanotubes, special types of graphene oxide, etc.), have been employed in the construction of AD immunosensors (Figure 2 and Figure 3), enabling a decrease in non-specific noise and amplification of the specific signal. Exploitation of these special nanomaterials has also enhanced robustness and miniaturized size of the analytical device (e.g., by employing arrays/ensembles of nanoelectrodes), thus providing the opportunity of point-of-care use of the AD immunosensors.

Due to their promising sensitivity/specificity analytical characteristics, along with a series of practical advantages offered in many cases, e.g., short analysis time (e.g., 30 min, [44]), miniaturized size, and/or capability of point-of-care analysis, immunosensors may eventually be commercialized and substantially contribute to prompt diagnosis of ADs. However, more research is required before AD immunosensors can be established as reliable clinical tools contributing to AD diagnosis. In our opinion, future research directions and main challenges include the following:

Multi-analyte detection might be a highly active research field in AD immunosensors. More specifically, efforts should be directed toward detection of a “disease-signature”-type panel of autoantibodies and/or protein biomarkers rather than a single analyte, which may greatly increase the clinical specificity of diagnosis. Recent developments in molecular biology and immunology, as well as further elucidation of disease-associated mechanisms/pathways, will help to identify/analyze such signature-type panels of biomarkers. On the other hand, fine-tuned technological/analytical improvements in immunosensors (e.g., further increase in analytical sensitivity) may help detect AD biomarkers that are present in patients’ samples prior to the manifestation of symptoms, but at very low concentrations. Progress in the above areas will further facilitate disease diagnosis/prognosis/follow-up, while it will promote the precision medicine-approach in ADs [92,93]. Moreover, cost and toxicity of all components of the AD immunosensors should decrease if large-scale production and wide applicability are to be achieved. The toxicity issue is important, especially in the case of future implantable immunosensors, which might serve, e.g., the in vivo monitoring of insulin along with glucose in patients with various types of diabetes [25].

Another issue associated with further improvement in the AD immunosensor field is the type of samples analyzed and the way through which these samples are collected. Actually, most of the samples reported to date are collected from human sera with blood drawing, or samples that are even more difficult to collect, such as cerebrospinal fluid (CSF) of MS patients, while tear analysis has been mentioned just once [80], at least to the best of our knowledge. Thus, more efforts should be directed toward the development of AD immunosensors suitable for analyzing samples that are easily obtained through non-invasive methods, e.g., urine or saliva.

Development of immunosensors for AD biomarkers that may allow the real-time sharing of the results obtained through a smartphone-based or a portable Wi-Fi-based signal-reading device would enhance the potential for various complex applications and home self-diagnosis, while it would enable exploitation/integration of the “new era” technological advances (Internet of things, artificial intelligence, and machine learning) in the field of AD immunosensors [34,94,95,96,97]. To the best of our knowledge, such an immunosensor has been proposed for the diagnosis of celiac disease [57].

In conclusion, besides their several advantages and strong points, AD immunosensors have not yet been commercialized and/or made available for clinical use. Integration/exploitation of the most recent technological advances, along with fine-tuned improvements in the design/construction of the analytical devices, in combination with thorough basic research to further elucidate AD pathophysiology mechanisms/pathways and new potential AD biomarkers, are considered necessary in order to eventually enable commercialization and wide clinical application of AD immunosensors. People of different specialties, including engineers, biochemists, immunologists, and medical doctors, can contribute to the achievement of this goal.

## Figures and Tables

**Table 1:** AD immunosensors that can detect specific autoantibodies.

Type of Signal Transduction	Immunoassay Principle—Use of Secondary Antibody	Autoantibody-Biomarker	Limit of Detection (LoD)/ Concentration Range	Autoimmune Disease	Biological Sample	References
Electrochemical (Cyclic voltammetry (CV) and electrochemical impedance spectroscopy (EIS))	Non-competitive, direct-type assay	Anti-oncostatin-M receptor autoantibodies	-	Systemic sclerosis (SSc)	Human serum from healthy individuals and SSc patients	[41]
Electrochemical (Voltammetry)	Non-competitive, direct-type assay	Anti-citrullinated peptide/protein autoantibodies (ACPAs)	15 pg mL^−1^/ 8–250 pg mL^−1^	Rheumatoid arthritis (RA)	Human serum (spiked)	[18]
Electrochemical (EIS)	Non-competitive, direct-type assay	ACPAs	0.82 IU ** mL^−1^/ 1–800 IU mL	RA	Human serum (spiked)	[42]
Optical (Spectral correlation interferometry—SCI)	Non-competitive, direct-type assay; a non-labeled secondary antibody was used	Anti-thyroglobulin (anti-TG) and anti-thyroid peroxidase (anti-TPO) autoantibodies	6 IU mL^−1^ (anti-TG) 1.7 IU mL^−1^ (anti-TPO)/ 6–400 IU mL^−1^ (anti-TG) 1.7–860 IU mL^−1^ (anti-TPO)	Autoimmune thyroid diseases	Patients’ serum	[46]
Piezoelectric quartz-crystal microbalance	Non-competitive, direct-type assay	Anti-phospholipase A2 receptor (anti-PL2R) autoantibodies	0.1 μg mL^−1^/ 0.5–100 μg mL^−1^	Primary membranous nephropathy (pMN)	Patients’ serum	[44]
Electrochemical (EIS)	Non-competitive, direct-type assay	Immunoglobulin M—rheumatoid factor (IgM-RF)	0.22 IU mL^−1^/ 10–200 IU mL^−1^	RA	Human serum (spiked)	[47]
Electrochemical (Voltammetry)	Non-competitive, direct-type assay; a labeled secondary antibody was used	Anti-tissue transglutaminase (anti-tTG) autoantibodies	1.8 ng mL^−1^/ 0.005–1 μg mL^−1^	Celiac disease (CD)	Serum from healthy individuals and CD patients	[50]
Electrochemical (Impedance spectroscopy and square wave voltammetry)	Non-competitive, direct-type assay	Autoantibodies on red blood cells (RBCs)	-	Autoimmune hemolytic anemia	Healthy and “sick” RBCs (i.e., RBCs from healthy and affected individuals)	[52]
Optical (Colorimetry)	Non-competitive, direct-type assay	IgM-RF	4.15 IU mL^−1^	RA	Human plasma (spiked)	[48]
Electrochemical (Electro-chemiluminescence—ECL)	Non-competitive, direct-type assay *; a labeled secondary antibody was used	Anti-myeloperoxidase (anti-MPO) autoantibodies	15.68 fg mL^−1^/ 50 fg mL^−1^–1 ng mL^−1^	Anti-neutrophil cytoplasm antibody-associated vasculitides	Human serum (spiked)	[55]
Electrochemical (Amperometry)	Non-competitive, direct-type assay *; a labeled secondary antibody was used	Anti-double-stranded DNA (anti-dsDNA) autoantibodies	8 μg mL^−1^	Systemic lupus erythematosus (SLE)	Patients’ serum	[56]
Electrochemical (Amperometry)	Non-competitive, direct-type assay; a labeled secondary antibody was used	Anti-tTG autoantibodies (IgG and IgA)	1.4 AU ** mL^−1^ (IgG) and 3.2 AU mL^−1^ (IgA)/ up to 30 AU mL^−1^ (IgG and IgA)	CD	Patients’ serum LOD: 3.2 AU **/mL (IgA), 1.4 AU/mL (IgG)	[57]
Electrochemical (Electro-chemiluminescence—ECL)	Non-competitive, direct-type assay	Anti-glutamate decarboxylase (anti-GAD) autoantibodies	0.10 ng mL^−1^/ 0.30–50 ng mL^−1^	Type-1 diabetes (T1D) or latent autoimmune diabetes in adult	Patients’ serum	[58]
Piezoelectric Quartz-crystal microbalance	Non-competitive, direct-type assay	Anti-TRIM21 and anti-TROVE2 autoantibodies	0.01 U ** mL^−1^ (anti-TRIM21) 0.005 U mL^−1^ (anti-TROVE2)/ 0.32–7.17 U mL^−1^ (anti-TRIM21) 0.07–1.46 U mL^−1^ (anti-TROVE2)	SLE	Serum from healthy individuals and SLE patients	[59]
Electrochemical (Amperometry)	Non-competitive, direct-type assay; a labeled secondary antibody was used	Anti-tTG autoantibodies	0.26 μg mL^−1^/ 0.26–6.9 μg mL^−1^	CD	Serum from CD patients	[60]
Electrochemical (Amperometry)	Non-competitive, direct-type assay; a labeled secondary antibody was used	Anti-tTG autoantibodies (IgA and IgG)	1.7 AU mL^−1^ (IgA) and 2.7 AU mL^−1^ (IgG)/ Up to 30 AU mL^−1^ (IgA and IgG)	CD	Serum from pediatric patients	[61]
Electrochemical (EIS)	Non-competitive, direct-type assay	Anti-Myelin Basic Protein (anti-MBP) autoantibodies	0.1495 ng mL^−1^/ 0.4875–2500 ng mL^−1^	Multiple sclerosis (MS)	Cerebrospinal fluid (CSF) and serum from relapsing/remitting MS patients	[62]
Electrochemical (CV)	Non-competitive, direct-type assay; a labeled secondary antibody was used	Anti-tTG autoantibodies	-	CD	Serum from CD patients	[63]
Electrochemical (Amperometry)	Non-competitive, direct-type assay *; a labeled secondary antibody was used	Anti-tTG autoantibodies	390 ng mL^−1^	CD	Serum from CD patients	[64]
Electrochemical (Amperometry)	Non-competitive, direct-type assay *; a labeled secondary antibody was used	ACPAs	-	RA	Serum from RA patients	[65]
Piezoelectric Quartz-crystal microbalance	Non-competitive, direct-type assay	ACPAs	-	RA	Serum from RA patients	[66]
Electrochemical (EIS)	Non-competitive, direct-type assay; a labeled secondary antibody was used	Anti-tTG autoantibodies	-	CD	Serum from CD patients	[67]
Piezoelectric Quartz-crystal microbalance	Non-competitive, direct-type assay	Anti-dsDNA autoantibodies	-	SLE	Serum from healthy individuals and patients with bronchial asthma and SLE	[68]
Optical (Surface plasmon resonance–SPR)	Non-competitive, direct-type assay	Anti-GAD autoantibodies	-	T1D	(Buffer)	[69]

* In the original papers, the immunocomplexes formed (antigen–autoantibody/analyte–secondary antibody) were characterized as “sandwich”. ** U: units; IU: international units; AU: arbitrary/antibody units.

**Table 2:** AD immunosensors that can detect protein biomarkers other than autoantibodies.

Type of Signal Transduction	Immunoassay Principle	Protein-Biomarker	LoD/ Concentration Range	Autoimmune Disease	Biological Sample	References
Optical (Multi Area Reflectance Spectroscopy—MARS)	Non-competitive, sandwich-type assay	Procalcitonin and interleukin-6 (IL-6)	2.0 ng mL^−1^ (PCT) and 0.01 ng mL^−1^ (IL-6)/ up to 100.0 ng mL^−1^ (PCT) and up to 10.0 ng mL^−1^ (IL-6)	Various inflammatory/autoimmune diseases	Human serum	[74]
Electrochemical (Cyclic voltammetry (CV) and electrochemical impedance spectroscopy (EIS))	Non-competitive, direct-type assay	Oncostatin-M receptor (sOSMR) protein	0.42 pg mL^−1^/ 0.005–500 pg mL^−1^	Systemic sclerosis (SSc)	Serum from healthy individuals and SSc patients	[41]
Electrochemical (Amperometry)	Non-competitive, sandwich-type assay	B cell activation factor (BAFF) and a proliferation-induced ligand (APRIL)	0.33 pg mL^−1^ (BAFF) and 16.4 pg mL^−1^ (APRIL) / 1.1–100 pg mL^−1^ (BAFF) and 0.05–20 ng mL^−1^ (APRIL)	Systemic lupus erythematosus (SLE)	Serum from healthy individuals and SLE patients	[76]
Electrochemical (Amperometry)	Non-competitive, sandwich-type assay	B cell activation factor (BAFF) and a proliferation-induced ligand (APRIL)	0.08 ng mL^−1^ (BAFF) and 0.06 ng mL^−1^ (APRIL) / 0.24–120 ng mL^−1^ (BAFF) and 0.19–25 ng mL^−1^ (APRIL)	SLE	Serum from SLE patients	[77]
Electrochemical (Amperometry)	Non-competitive, sandwich-type assay	CCL5 chemokine	40 pg mL^−1^/ 0.1–300 ng mL^−1^	Multiple sclerosis (MS)	Serum from healthy individuals and MS patients	[78]
Electrochemical (Amperometry)	Non-competitive, sandwich-type assay	IL-6	0.42 pg mL^−1^/ 0.97–250 pg mL^−1^	Rheumatoid arthritis (RA)	Human serum (spiked)	[22]
Electrochemical (Amperometry)	Non-competitive, sandwich-type assay	CXCL7 chemokine and MMP3 metalloproteinase	0.8 ng mL^−1^ (CXCL7) and 1.2 pg mL^−1^ (MMP3)/ 1–75 ng mL^−1^ (CXCL7) and 2.0–2000 pg mL^−1^ (MMP3)	RA	Serum from healthy individuals and RA patients	[79]
Electrochemical (EIS)	Non-competitive, direct-type assay	Tumor necrosis factor α (TNFα)	0.085 pg mL^−1^/ 1–25 pg mL^−1^	Various inflammatory/autoimmune diseases	Serum and tears from healthy individuals; cerebrospinal fluid (CFS) from patients undergone routine lumbar puncture	[80]
Electrochemical (Differential pulse voltammetry (DPV) and EIS)	Non-competitive, direct-type assay	Myelin Basic Protein (MBP) and Tau proteins	0.30 nM (MBP) and 0.15 nM (Tau)	MS	CSF and serum from MS patients	[82]
Electrochemical (Voltammetry)	Non-competitive, direct-type assay	Insulin	5 pM/ 5–200 pM	Diabetes types I and II	Serum from diabetic patients	[83]
Electrochemical (EIS)	Non-competitive, direct-type assay	Interleukin-12 (IL-12)	3.5 pg mL^−1^/ 0.1–500 pg mL^−1^	MS	Fetal bovine serum (FBS)	[84]
Electrochemical (Amperometry)	Non-competitive, direct-type assay	Macrophage migration inhibitory factor (MIF)	0.02 ng mL^−1^/ 0.03–230 ng mL^−1^	RA	Serum from RA patients	[85]
Electrochemical (EIS)	Non-competitive, direct-type assay	IL-12	<100 fM	MS	(Buffer)	[86]

## Data Availability

Not applicable.
